# Supplementary material for: Improvement of the Seminal Characteristics in Rams Using Agri-Food By-Products Rich in Phytomelatonin
Source: Animals (Basel). 2023 Mar 2;13(5):905. doi: 10.3390/ani13050905 (PMC10000078; doi:10.3390/ani13050905)

Figure S1: Rumen *in vitro* fermentation pattern (mL gas/g OM) of selected by-products: ■, pomegranate pomace; □, pomegranate peels; ▲, tomato pomace; ◆, grape pulp and ●, sunflower meal.

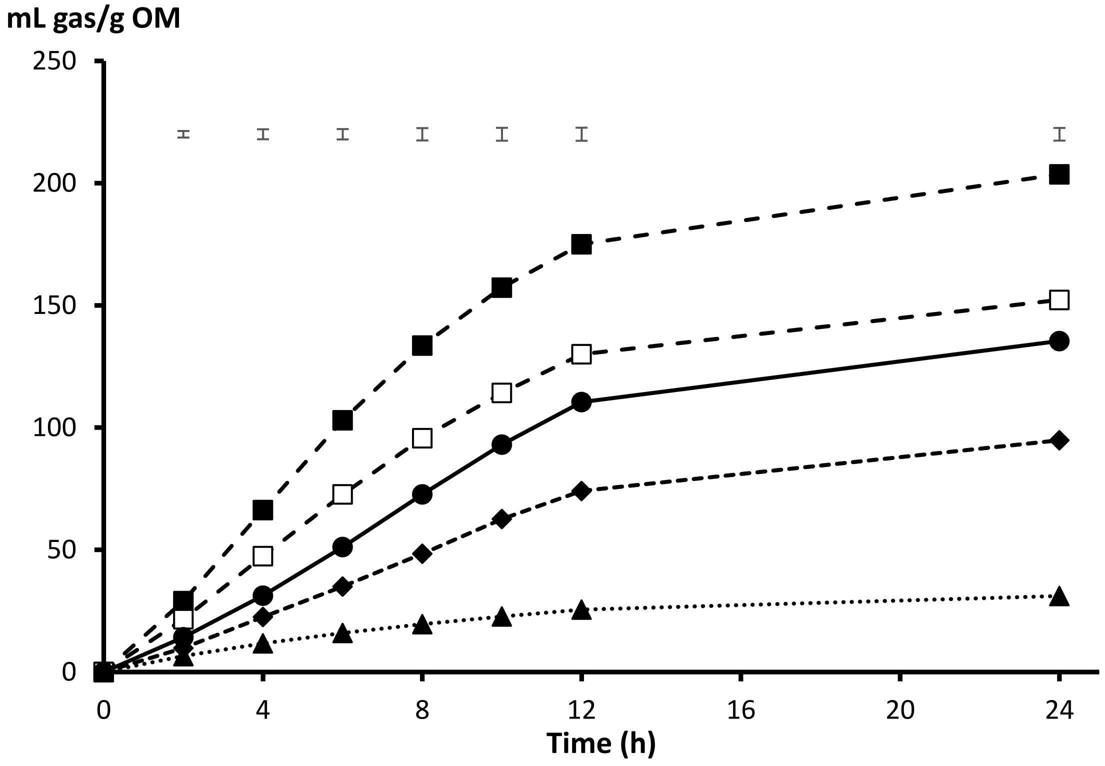

Supplement: Supplementary file 1 [file animals-13-00905-s001.zip › Figure S1.pdf]
