# Supplementary material for: Improvement of the Seminal Characteristics in Rams Using Agri-Food By-Products Rich in Phytomelatonin
Source: Animals (Basel). 2023 Mar 2;13(5):905. doi: 10.3390/ani13050905 (PMC10000078; doi:10.3390/ani13050905)
Supplement: Supplementary file 1 [file animals-13-00905-s001.zip › Table S1.pdf]

Table S1. Chemical composition (g/kg dry matter) of the selected by-products

	OM	CP	EE	NDF	ADF	ADL
Pomegranate pomace	963	104	19	241	178	78
Pomegranate peels	956	44	4	243	207	53
Tomato pomace	936	155	54	716	682	593
Grape pulp	894	126	49	293	335	201
Sunflower meal	942	295	9	454	351	159

OM, organic matter; CP, crude protein; EE, ether extract; NDF, neutral detergent fibre; DF, acid detergent fibre; ADL, acid detergent lignin.
