# Supplementary material for: Improvement of the Seminal Characteristics in Rams Using Agri-Food By-Products Rich in Phytomelatonin
Source: Animals (Basel). 2023 Mar 2;13(5):905. doi: 10.3390/ani13050905 (PMC10000078; doi:10.3390/ani13050905)
Supplement: Supplementary file 1 [file animals-13-00905-s001.zip › Table S2.pdf]

Table S2: *In vitro* ruminal and abomasal substrate digestibility (g/kg) of the selected by-products.

	Ruminal digestibility	Abomasal digestibility
Pomegranate pomace	640a	242a
Pomegranate peels	621a	93b
Tomato pomace	142b	43c
Grape pulp	344c	115b
Sunflower meal	513d	197d
SEM	9.3	7.6
P- value	<0.001	<0.001

SEM, standard error of means

Within each column, letters indicate significant differences ($P < 0.05$).
