# Supplementary material for: Improvement of the Seminal Characteristics in Rams Using Agri-Food By-Products Rich in Phytomelatonin
Source: Animals (Basel). 2023 Mar 2;13(5):905. doi: 10.3390/ani13050905 (PMC10000078; doi:10.3390/ani13050905)
Supplement: Supplementary file 1 [file animals-13-00905-s001.zip › Table S3.pdf]

Table 3.S. Chemical composition (g/kg dry matter): crude protein (CP), ether extract (EE), neutral detergent fibre (NDF) and acid detergent fibre (ADF) of the control and phytomelatonin-rich diets (n=2).

Diet	CP	EE	NDF	ADF
control	13.97 ± 0.04	4.07 ± 0.09	27.63 ± 0.23	18.26 ± 0.17
Phytomelatonin-rich	13.39 ± 0.07	3.50 ± 0.02	22.09 ± 0.01	12.31 ± 0.09
